# Effects of Heat-Not-Burn Cigarette Smoking on the Secretion of Saliva and Its Innate Immune System Components

**DOI:** 10.3390/healthcare11010132

**Published:** 2022-12-31

**Authors:** Yukihiro Mori, Mamoru Tanaka, Hana Kozai, Yuka Aoyama, Yukihiro Shigeno, Kiyoshi Hotta, Makoto Aoike, Hatsumi Kawamura, Masato Tsurudome, Morihiro Ito

**Affiliations:** 1 Graduate School of Life and Health Sciences, Chubu University, 1200 Matsumoto-cho, Kasugai 487-8501, Aichi, Japan; 2 Center for Nursing Practicum Support, Chubu University, 1200 Matsumoto-cho, Kasugai 487-8501, Aichi, Japan; 3 Department of Food and Nutritional Sciences, College of Bioscience and Biotechnology, Chubu University, 1200 Matsumoto-cho, Kasugai 487-8501, Aichi, Japan; 4 Department of Clinical Engineering, College of Life and Health Sciences, Chubu University, 1200 Matsumoto-cho, Kasugai 487-8501, Aichi, Japan; 5 The Fire Department Headquarters in Kasugai-City, 109-1 Umegatsubo-cho, Kasugai 486-0856, Aichi, Japan; 6 Department of Biomedical Sciences, College of Life and Health Science, Chubu University, Kasugai 487-8501, Aichi, Japan

**Keywords:** heat-not-burn tobacco, saliva, lactoferrin, lysozyme, antimicrobial proteins

## Abstract

Saliva and salivary antimicrobial proteins play important roles in the innate immunity, which prevents infections of orally invading bacteria and viruses. In this study, we compared the secretion rates of salivary lactoferrin (Lac) and lysozyme (Lys) in heat-not-burn (HNB) cigarette smokers and non-smokers. The analysis population for this study included 212 members of the fire department, including 32 HNB cigarette smokers, 17 paper cigarette smokers, 14 combined HNB and paper cigarette smokers, and 149 non-smokers. Salivary Lac and Lys concentrations were assessed using enzyme immunoassay. Saliva secretion was significantly lower among HNB cigarette smokers (*p* < 0.01) than among non-smokers. Accompanying this result, salivary Lac and Lys secretion rates were significantly lower among smokers, particularly HNB cigarette smokers, than among non-smokers (all *p* < 0.01). Our findings suggest a possible adverse effect of HNB cigarette on the amount of Lac and Lys released into the oral cavity.

## 1. Introduction

A heat-not-burn (HNB) smoking device contains an electronic device that heats tobacco leaves in a stick and the user inhales the aerosol produced instead of smoke [1]. In Japan, HNB cigarettes were launched in 2013. Since then, several products have been distributed and gained popularity. In Japan, the prevalence of typical HNB tobacco products increased from 0.3% in January–February 2015 to 0.6% in January–February 2016 to 3.6% in January–February 2017 [2]. On the other hand, the most recent survey in Japan in 2018 reported that estimates by smoking gear were “non-smokers” (male = 69.2%, female = 90.6%), “cigarette-only smokers” (male = 22.0%, female = 7.5%), “HNB cigarette smokers or dual users” (male = 7.2%, female = 1.4 %), and “other” (male = 1.7%, female = 0.5%) were reported [3]. It was also reported that the estimated number of HNB cigarette smokers in the Japanese population was 5.23 million (5%), and further that the proportion of HNB cigarette smokers was more than a quarter of the total tobacco user population [3]. The popularity of HNB cigarettes can be attributed to their flavors, which are designed to taste good to smokers, and the belief that they help people quit smoking [4]. However, compared with smoke from paper cigarettes, aerosols from mainstream HNB cigarettes contains the same level of nicotine and volatile compounds (acrolein and formaldehyde) and approximately thrice the level of acenaphthene (a polycyclic aromatic hydrocarbon) [5]. In recent years, adverse health effects from HNB cigarette smoking, such as acute eosinophilic pneumonia, have also been reported [6]. In addition, HNB cigarette use has been noted to have adverse effects on the cardiovascular system comparable to those of paper cigarettes, such as decreased vasodilation [7]. Furthermore, HNB cigarettes have been reported to exacerbate allergic diseases [8]. Thus, the adverse effects of HNB smoking on the respiratory and cardiovascular systems and allergic diseases have been noted in multiple ways, similar to those of cigarette smoking. Furthermore, nicotine has been considered a typical ingredient in conventional cigarettes that adversely affects immune function. Originally, smoking was thought to affect the human immune system and cellular and soluble inflammatory systems, and furthermore, smoking is thought to affect the entire cytokine network [9,10]. In terms of effects on innate immunity, it has been noted that salivary concentrations of Lactoferrin (Lac) [11] and Lysozyme (Lys) [12] may be decreased in smokers compared to nonsmokers. However, because these studies have not examined each smoking implement separately, and because HNB cigarettes have only been on the market for a few years, it is not clear as yet what kind of unique component or mechanism of HNB cigarettes affects the immune mechanism.

Saliva is a complex mixture of fluids secreted by the three major salivary glands—the parotid, submandibular, and sublingual glands—and numerous minor salivary glands located on the oral mucosa [13]. Saliva functions as an antimicrobial, eliminating pathogens and preventing the growth of microorganisms that can affect oral and systemic health [14,15,16].

Components of saliva associated with antimicrobial function include the antimicrobial proteins (AMPs): Lac and Lys. Both Lac and Lys are iron-binding glycoproteins secreted by serous glandular cells of the major and minor salivary glands. However, Lys is also secreted to a lesser extent by the gingival sulcus and salivary leukocytes [13]. They are important components of the innate immune system and prevent infectious diseases by acting as antimicrobials and antivirals [17,18].

However, to the best of our knowledge, the effects of HNB cigarette smoking on the secretion of salivary Lac and Lys are not known. Therefore, the aim of this study was to compare Lac and Lys secretion rates between non-smokers and HNB cigarette smokers cross-sectionally at a single time point. Lac and Lys have been noted as the most abundant and essential AMPs present in saliva [17], and have been used in multiple studies as a measure of AMPs in saliva [19,20]. Since the market for HNB cigarettes has increased in recent years, and this study is important from the perspective of public health and preventive care.

Firefighters, paramedics, and rescue workers working in fire stations located in a city in Japan were included in this study. Previous studies have reported exercise to be a factor that increases the salivary levels of Lac and Lys [19,21]. Therefore, the participants of this study may have relatively high innate immunity because they belong to a population that trains regularly and exercises heavily. However, we believe it is important to examine the innate immunocompetence of public service workers who, in the course of their duties, may be on the front line responding to persons with or suspected of having an infectious disease.

## 2. Materials and Methods

### 2.1. Study Participants

**Figure 1:**
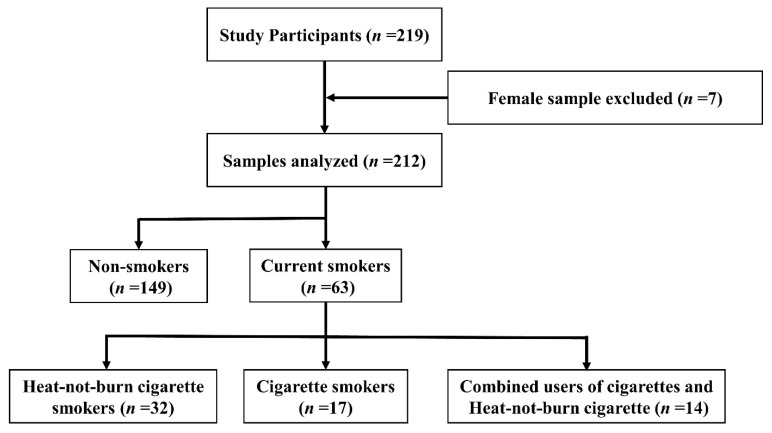
Flowchart in this study. 212 participants were included in this study, which comprised 32 HNB cigarette smokers, 17 paper cigarette smokers, 14 combined HNB and paper cigarette smokers, and 149 non-smokers.

The subjects of this study were firefighters, paramedics, and rescue workers working at five fire stations located in Kasugai, Japan. We recruited participants by distributing a research cooperation request form through the manager of each station. As shown in Figure 1, a total of 219 individuals participated in this study. In this study, seven female participants were all nonsmokers, so gender cannot be discussed as a confounding factor. Therefore, the analysis was narrowed down to 212 men: 32 HNB cigarette smokers, 17 paper cigarette smokers, 14 smokers who use both HNB and paper cigarettes, and 149 nonsmokers. In this study, “Paper cigarettes” were defined as conventional combustible cigarettes. Among the smokers, no one smoked electronic cigarettes. We collected saliva samples and administered a questionnaire on four separate occasions from 15 June 2021 to 24 June 2021, during the fourth outbreak of novel coronavirus infection in Japan. Therefore, we paid careful attention to hygiene and infection control while collecting the samples. Because dental findings could affect the results [22], those with periodontal disease were excluded [23]. Tooth loss was not observed in any of the analyzed subjects. Smokers were limited to those who had smoked continuously for at least 6 months.

### 2.2. Saliva Collection

Saliva was collected uniformly in a quiet room from 9:00 am to 12:00 am. Each participant was instructed not to eat, drink, brush their teeth, or smoke 60 min before and during saliva collection. In addition, a uniform method of stimulated saliva collection was used, based on a conventional method [24]. The importance of standardized conditions has been reported previously, and the use of stimulated saliva has been recommended to facilitate standardization [22,25]. Participants were asked to thoroughly rinse their oral cavity with distilled water three times per sitting. Next, participants were asked to swallow once the saliva accumulated in the oral cavity after 5 min of seated rest. Then, they were asked to chew a piece of tasteless sterile cotton (Salimetrics Oral Swab^TM^; Salimetrics, Carlsbad, CA, USA) once per second for 1 min while setting a timer. Newly secreted saliva was absorbed by the sterile cotton, which was then collected in a storage tube (Swab Storage Tube^TM^; Salimetrics). To separate saliva from the cotton, the storage tubes were centrifuged at 1400× *g* for 5 min and the volume of saliva was measured before being stored at −30 °C. The final volume of saliva was expressed as the saliva secretion rate (mL/min).

### 2.3. Lac and Lys Rating

The concentrations of Lac and Lys in saliva were assessed using enzyme-linked immunoassay (ELISA) with the AssayMax Human Lactoferrin ELISA kit (AssayPro, St. Charles, MO, USA) and AssayMax Lysozyme ELISA kit (AssayPro), respectively, according to the manufacturer’s instructions [19,26].

In this study, the secretion rates (μg/min) of Lac and Lys were used as evaluation indices, which were calculated from the absolute concentration of each marker (μg/mL) and the saliva secretion rate (mL/min).

### 2.4. Survey

Information on the characteristics of each participant was collected using a questionnaire. In addition to, participants responded to questions regarding smoking habits as well as age group, gender, and history of chronic disease.

### 2.5. Statistical Analysis

Numerical data are presented as the median (Interquartile range: IQR). The Mann–Whitney U test was used to compare each marker between non-smokers and smokers. The Kruskal–Wallis test was used to compare all four groups, and multiple comparisons were verified using Bonferroni’s method. Statistical significance level was considered as *p* < 0.05. IBM SPSS Statistics version 27 (IBM Corp., Armonk, NY, USA) was used for statistical analysis.

## 3. Results

### 3.1. Study Participants

The age group, gender, and smoking habits of the participants are shown in Table 1. With regard to a history of chronic diseases, seven individuals had hypertension (3.3%), seven individuals had hyperlipidemia (3.3%), one individual had diabetes mellitus (0.5%), and one individual had rheumatoid arthritis (0.5%).

### 3.2. Saliva Secretion Rate

As shown in Table 2, the saliva secretion rate of smokers, with a median (IQR) value of 1.0 (0.7–1.4) mL/min, was significantly lower than that of non-smokers, with a median (IQR) value of 1.4 (0.9–2.2) mL/min (*p* < 0.001). It was worth noting that the saliva secretion rate of HNB cigarette smokers (1.0 (0.7–1.5) mL/min) was significantly lower than that of non-smokers (*p* = 0.010), while the difference between cigarette smokers and non-smokers was less significant (*p* = 0.045).

On the other hand, we found no noteworthy significant difference in the concentrations of salivary Lac and Lys either between non-smokers and smokers, regardless of smoking habit groups (Table 2).

### 3.3. Lac Secretion Rate

**Figure 2:**
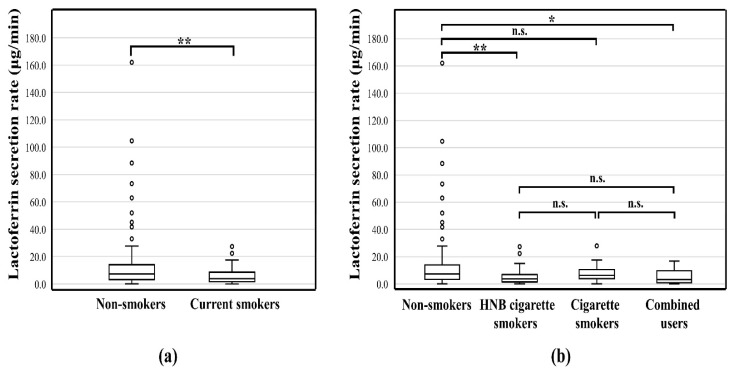
(**a**) Comparison of the rate of Lactoferrin secretion in non-smokers and smokers; (**b**) Comparison of the rate of Lactoferrin secretion by different smoking devices; **, *p* < 0.01; *, *p* < 0.05; n.s., no significant difference; HNB, Heat-not-burn.

The Lac secretion rate of smokers (3.9 (1.7–8.5) μg/min) was significantly lower (*p* < 0.01) than that of non-smokers (7.3 (3.3–13.8) μg/min) (Figure 2a). The Lac secretion rate of HNB cigarette smokers (3.6 (1.5–6.9) μg/min) was significantly lower than that of non-smokers (*p* < 0.01). Compared with the non-smoking group, the combined (HNB and cigarette smokers) group showed lower Lac secretion rate (3.2 (1.0–9.3) μg/min), but the difference was weakly significant (*p* < 0.05) (Figure 2b).

### 3.4. Lys Secretion Rate

**Figure 3:**
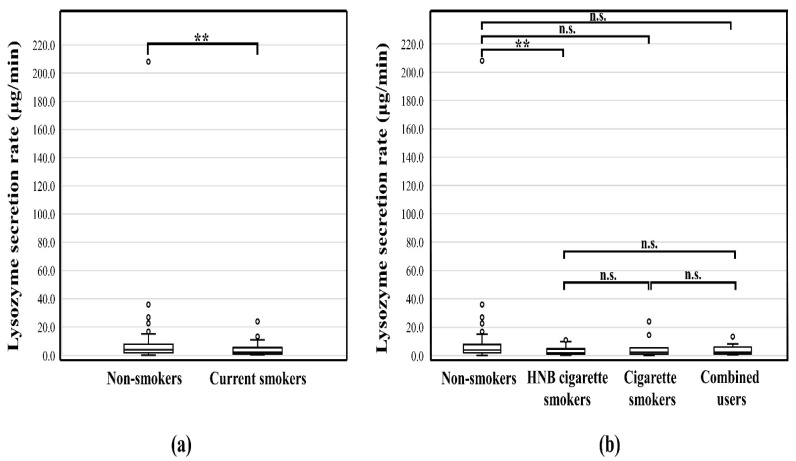
(**a**) Comparison of the rate of lysozyme secretion in non-smokers and smokers; (**b**) Comparison of the rate of lysozyme secretion by different smoking devices; **, *p* < 0.01; n.s., no significant difference; HNB, Heat-not-burn.

The Lys secretion rate of smokers (2.2 μg/min (1.1–5.5) μg/min) was significantly lower (*p* < 0.01) than that of non-smokers (4.0 μg/min (1.9–7.7) μg/min) (Figure 3a). Specifically, the Lys secretion rate of HNB cigarette smokers (2.1 μg/min (1.1–4.5) μg/min) was significantly lower than that of non-smokers (*p* < 0.01) (Figure 3b).

## 4. Discussion

This study suggested that HNB cigarette smokers may have lower saliva and Lac and Lys secretion rates compared to non-smokers. Several previous studies have reported a reduction in saliva secretion due to smoking [27,28,29]. Additionally, the salivary concentration of Lac [11] and Lys [12] is lower in smokers than in non-smokers. However, these studies have not been comparatively verified for different smoking devices. In a study in Poland, reduction of Lys levels was verified for smokers, including electronic cigarette smokers [23]. However, the study did not include HNB cigarette smokers, who are prevalent in Japan. Therefore, to the best of our knowledge, the results of the present study have not been presented previously.

In addition, to the best of our knowledge, most of the previous studies reporting the association between smoking habits and Lac and Lys levels have used the concentration of each marker as an assessment index. However, several studies have pointed out that the availability of AMPs in saliva at the oral surface may play an important role in mucosal immunity and that reporting the secretion rate of each marker may be a more appropriate indicator than concentration alone [30,31]. This is because low saliva production may reduce the absolute amounts of AMPs such as Lac and Lys released into the oral cavity. Therefore, the strength of this study is that the secretion rate of Lac and Lys in saliva was used as an indicator for evaluation. We could not find statistical difference in Lac and Lys concentrations between smokers and nonsmokers in this study unlike the results of previous studies [11,12]. In all likelihood, this discrepancy could have arisen due to our selective sampling: that is, choosing fire department personnel. In any case, our current study has indicated at least that smoking HNB cigarettes decreases the rate of Lac and Lys secretion in these peoples.

However, to date, the scientific evidence on the mechanism by which smoking affects the secretion levels of Lac and Lys has not been fully elucidated. Saliva is secreted by each salivary gland, large and small [13], and AMPs such as Lys and Lac are also derived from salivary glands [32]. Not to mention the negative effects of smoking on the body as a whole. The salivary glands, which are initially exposed to cigarette smoke inhaled through the mouth, may also be targeted by the effects of smoking [33]. Tobacco smoke contains thousands of components in its gaseous and particulate stages [34]. Nicotine intake causes morphological and functional changes in the salivary glands [35]. In addition, nicotine may adversely affect the synthesis and secretion of Lac and Lys [36]. A study comparing chemicals in mainstream smoke in HNB cigarettes and cigarette papers found that nicotine concentrations in mainstream smoke of some HNB cigarette products and paper cigarettes were almost identical [37]. Therefore, even nicotine ingestion during HNB cigarette smoking may hypothetically have an adverse effect on the salivary glands, reducing the rate of Lac and Lys secretion.

In the present study, a significant difference in Lac and Lys secretion rate was observed between the HNB cigarette smoker and non-smoker group. However, there was no significant difference between the non-smoker group and the cigarette smokers. Based on these results, the possible involvement of toxic substances other than nicotine specific to HNB cigarettes, which do not occur frequently in paper cigarettes, must be considered. Previous studies have reported that the total amount of chemicals produced by HNB cigarettes is not significantly different from that of paper cigarettes; in fact, some chemicals are produced by HNB cigarettes are not found in paper cigarettes [38].

In our study, about half of the smokers were HNB cigarette users, which differs from the general rate of use in Japan [2,3]. Therefore, further research is needed to interpret the results of this study as representative of the general population. In addition to that, in this study, we had set the selection criterion as smokers who had been smoking for a certain period of time, but information on the exact duration of smoking dependence and the amount of smoking were not available. Therefore, we were not able to perform a statistical analysis that considered these factors as covariates. Owing to the cross-sectional nature of this study, we did not identify variation over time. Furthermore, since all of the subjects analyzed in this study were male, the effect of gender must be examined in the future. In addition, previous studies have reported an association between circadian rhythm and saliva production [39]. The fire department personnel in our study may have a different circadian rhythm than the general population due to their irregular work regime. However, the present study did not include detailed sleep conditions in the analysis. It has also been noted that fluid loss affects salivary flow [40] and may affect the concentration and secretion rate of AMP in saliva. In the present study, we were not able to determine in detail the dehydration tendency of the participating fire troopers.

Nevertheless, the present study is of great importance for preventive medicine to show the possible adverse effects of the recent popularity of HNB cigarette smoking on innate immunity. The antimicrobial [41,42] and antiviral [43,44] actions of Lac and Lys have been validated in multiple studies and their importance as innate immune system components has long been noted. In addition, in vitro data have recently been reported on the potential antiviral immune response of Lac in the prevention of severe acute respiratory syndrome coronavirus 2 infection [45]. With emerging and re-emerging infectious diseases spreading globally, we believe that the results of this study will contribute to raising new public health issues related to HNB cigarettes.

## 5. Conclusions

Knowledge of the health hazards of HNB cigarette smoking is limited. Despite this, the number of users is increasing and is a public health issue. The present study suggests a possible adverse effect of HNB cigarette smoking on the secretion rate of salivary Lac and Lys. Our findings present a potential new health hazard to innate immunity posed by HNB cigarette smoking.

## Figures and Tables

**Table 1:** Participant characteristics.

	*n*	%
Age groups		
20–29	67	31.6
30–39	75	35.4
40–49	52	24.5
>50	18	8.5
Sex		
Male	212	100.0
Smoking habit		
Non-smokers	149	70.3
Current smokers	63	29.7
HNB cigarette smokers	32	51.0
Cigarette smokers	17	27.0
Combined users of cigarettes and HNB cigarette	14	22.0

HNB: Heat-not-burn.

**Table 2:** Saliva secretion rates and concentrations of Lac and Lys.

	Saliva Secretion Rate (mL/min)	*p*-Value ^†^	Lac Concentration (μg/mL)	*p*-Value ^†^	Lys Concentration (μg/mL)	*p*-Value ^†^
Non–smokers	1.4 (0.9–2.2)		4.5 (2.5–10.2)		2.8 (1.5–4.8)	
Current smokers	1.0 (0.7–1.4)	<0.001	4.3 (2.1–7.1)	0.094	2.4 (1.1–3.9)	0.243
HNB cigarette smokers	1.0 (0.7–1.5)	0.010	3.6 (2.7–6.1)	0.054	2.3 (1.2–3.4)	0.152
Cigarette smokers	1.1 (0.6–1.3)	0.045	6.0 (3.9–8.8)	0.618	3.2 (1.0–5.3)	0.850
Combined users	1.1 (0.7–1.5)	0.100	3.5 (1.0–7.5)	0.125	2.6 (1.0–4.5)	0.738

All numerical data are medians (Interquartile range: IQR). HNB, Heat-not-burn; Lac, Lactoferrin; Lys, Lysozyme; ^†^, comparison with Non-smokers.

## Data Availability

Not applicable.
